# Trends of *Plasmodium falciparum* prevalence in two communities of Muheza district North-eastern Tanzania: correlation between parasite prevalence, malaria interventions and rainfall in the context of re-emergence of malaria after two decades of progressively declining transmission

**DOI:** 10.1186/s12936-018-2395-1

**Published:** 2018-07-06

**Authors:** Deus S. Ishengoma, Bruno P. Mmbando, Celine I. Mandara, Mercy G. Chiduo, Filbert Francis, Wilbert Timiza, Hellen Msemo, Agnes Kijazi, Martha M. Lemnge, Mwelecele N. Malecela, Robert W. Snow, Michael Alifrangis, Ib C. Bygbjerg

**Affiliations:** 1 Tanga Research Centre, National Institute for Medical Research, Tanga, Tanzania; 2 Tanzania Meteorological Agency, Dar es Salaam, Tanzania; 3 National Institute for Medical Research, Headquarters, Dar es Salaam, Tanzania; 4 Kenya Medical Research Institute/Wellcome Trust Research Programme, Nairobi, Kenya; 5 Centre for Tropical Medicine and Global Health, Nuffield Department of Clinical Medicine, University of Oxford, Oxford, UK; 6 Centre for Medical Parasitology, Department of Immunology and Microbiology, University of Copenhagen, Copenhagen, Denmark; 7 Section of Global Health, Department of Public Health, University of Copenhagen, Copenhagen, Denmark

## Abstract

**Background:**

Although the recent decline of malaria burden in some African countries has been attributed to a scale-up of interventions, such as bed nets (insecticide-treated bed nets, ITNs/long-lasting insecticidal nets, LLINs), the contribution of other factors to these changes has not been rigorously assessed. This study assessed the trends of *Plasmodium falciparum* prevalence in Magoda (1992–2017) and Mpapayu (1998–2017) villages of Muheza district, North-eastern Tanzania, in relation to changes in the levels of different interventions and rainfall patterns.

**Methods:**

Individuals aged 0–19 years were recruited in cross-sectional surveys to determine the prevalence of *P*. *falciparum* infections in relation to different malaria interventions deployed, particularly bed nets and anti-malarial drugs. Trends and patterns of rainfall in Muheza for 35 years (from 1981 to 2016) were assessed to determine changes in the amount and pattern of rainfall and their possible impacts on *P. falciparum* prevalence besides of those ascribed to interventions.

**Results:**

High prevalence (84–54%) was reported between 1992 and 2000 in Magoda, and 1998 and 2000 in Mpapayu, but it declined sharply from 2001 to 2004 (from 52.0 to 25.0%), followed by a progressive decline between 2008 and 2012 (to ≤ 7% in both villages). However, the prevalence increased significantly from 2013 to 2016 reaching ≥ 20.0% in 2016 (both villages), but declined in the two villages to ≤ 13% in 2017. Overall and age specific *P. falciparum* prevalence decreased in both villages over the years but with a peak prevalence shifting from children aged 5–9 years to those aged 10–19 years from 2008 onwards. Bed net coverage increased from < 4% in 1998 to > 98% in 2001 and was ≥ 85.0% in 2004 in both villages; followed by fluctuations with coverage ranging from 35.0 to ≤ 98% between 2008 and 2017. The 12-month weighted anomaly standardized precipitation index showed a marked rainfall deficit in 1990–1996 and 1999–2010 coinciding with declining prevalence and despite relatively high bed net coverage from 2000. From 1992, the risk of infection decreased steadily up to 2013 when the lowest risk was observed (RR = 0.07; 95% CI 0.06–0.08, P < 0.001), but it was significantly higher during periods with positive rainfall anomalies (RR = 2.79; 95% CI 2.23–3.50, P < 0.001). The risk was lower among individuals not owning bed nets compared to those with nets (RR = 1.35; 95% CI 1.22–1.49, P < 0.001).

**Conclusions:**

A decline in prevalence up to 2012 and resurgence thereafter was likely associated with changes in monthly rainfall, offset against changing malaria interventions. A sustained surveillance covering multiple factors needs to be undertaken and climate must be taken into consideration when relating control interventions to malaria prevalence.

**Electronic supplementary material:**

The online version of this article (10.1186/s12936-018-2395-1) contains supplementary material, which is available to authorized users.

## Background

Sub-Saharan Africa has witnessed an epidemiological transition in the distribution and intensity of malaria transmission since 2000, with a remarkable decline of malaria burden up to 2015 and a resurgence reported in 2016 [1, 2]. The declining burden of malaria has often been directly attributed to scale-up of interventions including vector control and changing anti-malarial treatment policies [1–4]. However, malaria is influenced by a complex array of environmental, ecological and climate factors and a desire to attribute the changes to intervention may mask this complex interplay of biotic and abiotic factors [2]. A detailed analysis of factors affecting malaria transmission requires congruent temporal data over long periods [2]. However, such long-term data which would support stratification of disease burden and setting up targeted control strategies with most impact is commonly lacking and decisions are often made based on limited updated evidence and high levels of uncertainty. Thus, malaria control strategies and interventions have frequently been implemented without proper stratification of current malaria transmission which would have presented a more balanced picture of malaria transmission intensity and disease burden in specific areas, and provide better evidence for more targeted interventions with higher impacts [5]. Malaria control in Tanzania is of no exception: a paper examining the distribution of insecticide-treated nets (ITNs) showed clustering of ITNs which generally did not reflect patterns of transmission and in some places even an inverse distribution vs. intensity of transmission [6].

Longitudinal studies in Kenya [7, 8], Senegal [9] and Guinea Bissau [10] have all demonstrated that the changing epidemiology of infection and disease cannot be easily explained by changing coverage of interventions such as vector control alone. While these studies all demonstrate an overall decline in malaria, in Kenya and Guinea Bissau, resurgent risks were documented after 2012 and this was consistent with resurgent risks during the same period in Zimbabwe [11] and Mozambique [12, 13]. Furthermore, recent reports have also showed a resurgence of malaria in many countries particularly in 2016, whereby malaria cases increased by over 6 million compared to 2015 [1, 2].

Despite scarcity of long-term data on malaria prevalence across Tanzania, the consensus that the burden of malaria has declined significantly since 2000 is most likely valid [14, 15]. However, the decline has been less dramatic in some of the historically high burden northwestern and southern areas of Tanzania [14–16]. In the village of Nyamisati, in the Rufiji River Delta, Pwani region malaria prevalence was > 70% in 1985 and had declined to 5% in 2010 [17]. In Korogwe district, Tanga region, the prevalence *of Plasmodium falciparum* declined from 78% in 2003 to 13% in 2008 in the lowland village and from 25 to 3% in the highland village [18]. Finally, a study conducted in the two villages of Magoda and Mpapayu in Muheza district, also of Tanga region showed high malaria prevalence (> 68%) from 1992 to 1999 which was followed by a moderate decline from 2000 to 2004 and then, a significant decline from 2008 to 2012 [19]. The present study is an extension of the latter study where the aims were to describe the most recent trends of *P*. *falciparum* prevalence in the two villages and examine plausible factors that may have contributed to the changing transmission intensity of *P*. *falciparum* between 1992 and 2017.

## Methods

### Study sites

The data used in this analysis was obtained from studies conducted in two villages of Magoda and Mpapayu in Muheza district. It is an extension of the analysis which covered studies implemented from 1992 to 2004 where different types of studies were undertaken in these villages including drug efficacy trials, bed net effectiveness trials and parasitological surveillance through cross-sectional surveys (CSS) [20–22]. With dramatic changes of malaria epidemiology observed in mid 2000s, a series of CSS were initiated from 2008 to 2017 to document changes in malaria transmission and trends of malaria burden and identify possible factors associated with and possibly driving the current epidemiological changes. Data from studies conducted between 1992 and 2004, and CSS done between 2008 and 2012 have been published earlier [19]. In the present study, these data have been extended to include data from CSS performed between 2013 and 2017 and as well combined with rainfall data spanning the years 1981–2016.

### Recruitment of participants and data collection

Although the CSS conducted before 2008 involved individuals of all age groups, for comparison reasons the current analysis focused only on those aged 0–19 years. For the CSS conducted from 2008 onwards, 250 individuals were randomly selected in each study village as previously described [23]. From 1992 to 2004, the CSS were conducted either before/during short (August–December) or long (April/June) rainy seasons while the CSS undertaken from 2008 were done during or after the long rains between May and June. Recruitment, examination and enrolment of study participants involved obtaining demographic details, physical examination and assessment of splenomegaly [19]. Blood samples were collected from each of the study participants by venous bleeding or finger prick for parasitological examination and other laboratory analyses. Thick and thin blood smears were prepared and dried in the field, and later brought to the laboratory for further processing.

### Laboratory analysis

Blood smears were stained using 3% Giemsa solution for 45 min and examined at a magnification of 1000× to detect parasite species and to determine parasitaemia. Reporting, quantification and quality control of microscopic examination of blood smears were performed as described elsewhere [23, 24].

### Control interventions against malaria

Between 1992 and 2001, chloroquine was used for the treatment of uncomplicated malaria while sulfadoxine/pyrimethamine (SP) and amodiaquine (AQ) were also used before and after policy changes in 2001 and until 2006 when they were withdrawn. In November 2006, artemether–lumefantrine (AL) replaced SP and has been the first-line drug for the treatment of uncomplicated falciparum malaria without parasitological confirmation (from January 2007) or with confirmed parasitological test by malaria rapid diagnostic tests (RDTs) from 2012. A weekly mobile clinic was introduced in Magoda in 1994 and Mpapayu in 1997; whereby during each visit, febrile individuals were tested for malaria by microscopy and treated with either AQ or SP. In 2004, the mobile clinic was replaced with a dispensary which was constructed by the project [19]. From 2008, a new surveillance was initiated whereby all participants (6 months to 19 years old) were tested with RDTs and in the CSS conducted in 2008 and 2009, only those with positive test results and other symptoms of uncomplicated malaria were treated with AL as described by Ishengoma et al. [19, 23]. From 2010, in accordance with the national guidelines [25], both symptomatic and asymptomatic individuals with positive RDT results, seen during the CSS were treated with AL.

### Bed-coverage and variation in malaria vectors

In December 1998, each sleeping bed in Magoda received a permethrin insecticide-treated net (ITN), while in Mpapayu, deltamethrin-treated nets were distributed in March 2001; all ITNs were re-impregnated with the same insecticides twice a year until 2003 [20]. From 2004, long-lasting insecticidal nets (LLINs) replaced conventional ITNs and were distributed free of charge to all household members through the National Universal Bed net Coverage Campaign and other national programmes including the discounted voucher scheme for pregnant women and infants, and school nets malaria programme (R. Mandike and S. Mkude, personal communication). There have been no attempts to use indoor residual house-spraying and larviciding in either village.

Few studies were conducted between 1992 and 2004 to monitor mosquito vectors in the study villages and showed that *Anopheles gambiae* was the main malaria vector in the community, together with *Anopheles funestus* and a low proportion of *Anopheles arabiensis* (Amani Centre, unpublished reports). It was also shown that *An. gambiae* sensu stricto was the main malaria vector in Muheza and other parts of north-eastern Tanzania followed by *An. funestus* until midi 2000’s when *An. arabiensis* started to become prominent [26]. Other studies have shown an increase in vector resistance to commonly used pyrethroids insecticides (permethrin, deltamethrin and lamda cyhalothrin) in Muheza as well as DDT in other parts of Tanzania in 2011 compared to 2004 and 2010 [27, 28]. However, no vector surveillance has been undertaken in the study villages in recent years.

### Rainfall data

Rainfall data for Muheza district spanning a period of 35 years (from 1981 to 2016) was obtained from the Tanzania Meteorological Agency (TMA), covering weather stations around Muheza town (situated about 10 km from Magoda and Mpapayu villages). Monthly total rainfall data was used in the analysis and for months with missing data, the gaps were filled with satellite-generated data.

### Ethical considerations

The studies which provided parasitological and clinical data for this paper were approved by the Medical Research Coordinating Committee (MRCC) of the National Institute for Medical Research (NIMR). Permission to conduct the study was sought from the regional and district medical officers of Tanga and Muheza, respectively; and from leaders of the two villages. Meetings were held with all community members to obtain their acceptance before the project started. Verbal and written informed consent was sought from patients or parents/guardians in case of children. Further meetings were held in each village to give feedback including results of previous surveys and discuss the study plans with community members. A written report of the previous survey was also given at each meeting.

### Data analysis

Data collected in previous studies were managed as described earlier [19], while the data collected from 2008 were double entered in Microsoft Access database followed by validation, cleaning and analysis using STATA version 13 (STATA Corp Inc., TX, USA). Rainfall data covering a period of 1981–2016 was managed with Microsoft Excel, and later transferred to STATA and R Statistical software [29] for analysis. The analysis involved comparison of parasite prevalence in order to test for the differences across years and between the two villages. The analysis was also conducted for different age groups of under-fives, individuals aged 5–10 years old and those aged ≥ 10 years as previously described [19]. The mean monthly rainfall was calculated from data collected by weather stations around Muheza town. Rainfall data derived from Rain-Gauge observations merged with satellites rainfall estimates (for months with missing rainfall data) were used to generate a complete dataset, and the results were summarized in tabular and line plots. Rainfall estimates derived from satellite data have been shown to have better quality and cover up or improve rainfall data availability over areas with sparse network of rainfall observations [30–32]. To determine the effect of rainfall on the risk of malaria infection, the 12-month weighted anomaly standardized precipitation (WASP) index (baseline = January 1981) was estimated as previously described [33, 34]. Modelling of number of malaria positive cases against the total number of surveyed individuals (as offset) was done using Poisson model, with bed net (ITNs/LLINs) ownership/coverage and rainfall anomaly, year of survey and study village as covariates. Robust standard errors for the parameter estimates were used to control for possible violation of the distribution with an assumption that the variance equals the mean and heteroskedasticity.

## Results

### Prevalence of *Plasmodium falciparum* infections in individuals aged 0–19 years in Magoda (1992–2017) and Mpapayu village (1998–2017)

**Fig. 1:**
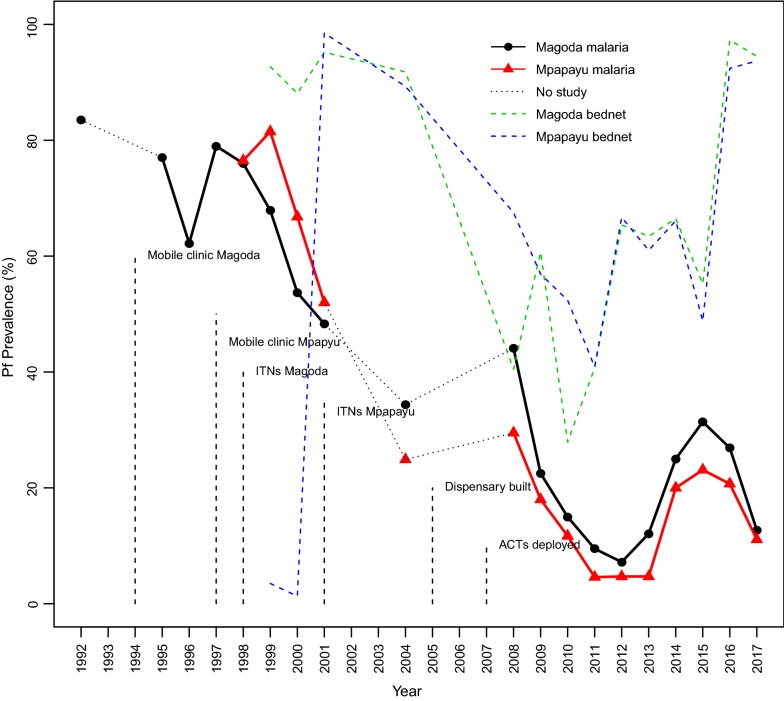
Prevalence of *Plasmodium falciparum* detected by microscopy in individuals aged 0–19 years and different interventions deployed in the two villages of Muheza district from 1992 to 2017 in Magoda and 1998 to 2017 in Mpapayu. Pf: *P. falciparum*

For parasite prevalence data between 1992 and 1997 in Magoda village, only summaries obtained from previous reports were available while for both villages between 1998 and 2017, a total of 9841 individuals aged 0–19 years were sampled in the 15 CSS (details shown in Additional file 1). Between 1992 and 1999, the prevalence of *P. falciparum* infections in Magoda village showed a moderate decline, except for a marked drop to 62.2% in 1996 (Fig. 1). In 1999, the prevalence was 67.7% in Magoda village while it was 81.5% in Mpapayu, and declined to 53.7 and 66.8% in 2000 in the two villages, respectively. From 2000 to 2004, a sharp decline occurred (to 34.4 and 24.9%, in Magoda and Mpapayu villages, respectively), followed by a slight increase in 2008 to 44.1% in Magoda and 29.5% in Mpapayu. Between 2009 and 2012, there was a sharp and progressive decline to a prevalence of only 7.2 and 4.7% in 2012 in Magoda and Mpapayu villages, respectively. Finally, the following years (from 2013 to 2015) had a significant increase of parasite prevalence (reaching 31.4 and 23.1% in 2015 in Magoda and Mpapayu villages, respectively) while in the survey of 2016, *P*. *falciparum* prevalence was at a similar plateau as in 2015 but thereafter declined to 13.3% in Magoda and 11.1% in Mpapayu in 2017 (Fig. 1).

### Age-specific prevalence of *P. falciparum* infections in Magoda (1992–2017) and Mpapayu village (1999–2017)

**Fig. 2:**
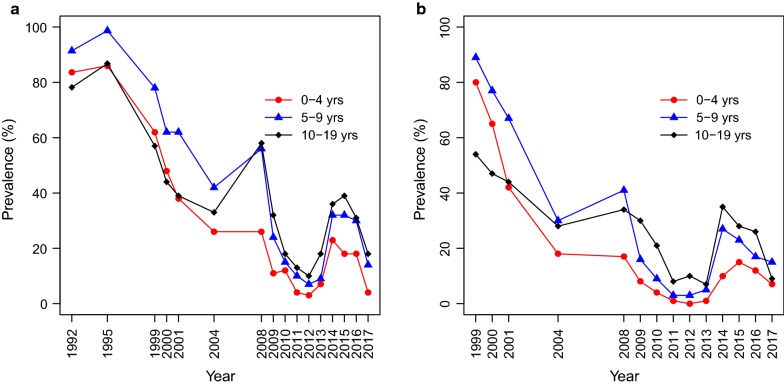
Age specific prevalence of *Plasmodium falciparum* detected by microscopy in the two villages of Magoda (**a**) and Mpapayu (**b**) in Muheza district from 1992 to 2017

Among under-fives in Magoda village, the highest prevalence of *P. falciparum* infections (86.0%) was reported in 1995 and it declined to 48.1% in 2000. Thereafter, the prevalence among under-fives continued to decline reaching approximately 26% in 2004 and 2008, and then declined to the lowest level of 3.3% in 2012 (Fig. 2). From 2013, parasite prevalence among under-fives in Magoda increased to 6.8% and reached the highest level (23.4%) in 2014 and started to decline with a prevalence of 6.6% in 2017. In Mpapayu village, the highest parasite prevalence among under-fives was 80.2% in 1999 with a slight decline to 77.2% in 2000 and a further drop to approximately 17–19% in 2004 and 2008. In 2012, no child aged less than 5 years from Mpapayu had malaria parasites. This was followed by an increase in prevalence reaching the highest of 14.8% in 2015 and then declined to 7.0% in 2017 (Fig. 2).

Children aged 5–9 years in Magoda (from 1992) and Mpapayu (from 1999) had the highest prevalence of *P. falciparum* infections compared to other age groups up to 2004 in Magoda and 2008 in Mpapayu; with the highest prevalence of 98.7 and 89.4% in Magoda in 1995 and in Mpapayu in 1999, respectively. A similar decline (as in under-fives) was also observed in this age group with the lowest prevalence in 2012 (6.7% in Magoda and 3.2% in Mpapayu); but with an increase in the prevalence in both villages reaching the highest prevalence of 32.3% in 2015 in Magoda and 26.9% in Mpapayu in 2014. A decline in prevalence occurred (among children aged 5–9 years) in 2016 and 2017 in Magoda and from 2015 in Mpapayu with the lowest of approximately 15.0% in 2017 in the two villages (Fig. 2). Among children aged 10–19 years, a declining but generally lower prevalence was observed compared to other groups between 1992 and 2000 in Magoda, and 1999–2000 in Mpapayu. Parasite prevalence was 43.9 and 46.8% in 2000, with a further decline to 32.8 and 27.5% in 2004 in Magoda and Mpapayu, respectively. Generally, this age group had lower *P. falciparum* prevalence compared to those aged 5–9 years in the first years of the study, however, from 2008 to 2009 onwards (in both villages), the opposite situation was observed whereby the highest prevalence shifted to this age group except in Mpapayu in 2017 (Fig. 2 and Additional file 2).

### Bed net coverage in Magoda and Mpapayu villages between 1999 and 2017

In Magoda village, coverage (ownership) of bed nets (ITNs/LLINs) increased from less than 4% in 1998 to 92.7% in 1999 and reached the highest level of 95.2% in 2001 (Fig. 1). In Mpapayu village however, bed net coverage was less than 4% up to 2000 but increased to 98.5% in 2001. The coverage of ITNs/LLINs was at the lowest level (40.5%) in Magoda village in 2008, but increased to 60.7% in 2009 and then declined to 40.7% in 2011, followed by an increase to 82.2% in 2012 (Fig. 1). A similar pattern was observed in Mpapayu between 2008 and 2012. The coverage decreased from 63.3 to 54.0% and 61.0 to 49.0% between 2013 and 2015 in Magoda and Mpapayu, respectively; while very high coverage of bed nets (> 92.0%) was reported in both villages in 2016 and 2017 (Fig. 1).

### Rainfall pattern in Muheza town from 1981 to 2016

**Fig. 3:**
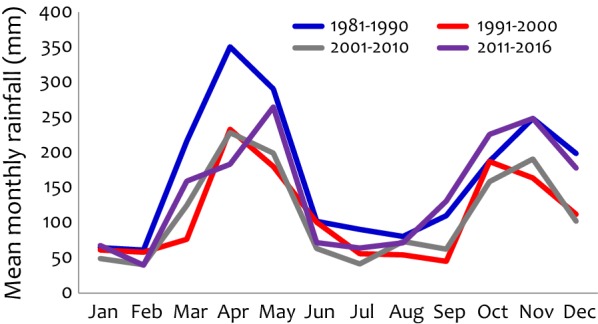
Mean monthly rainfall in Muheza from 1981 to 2016

The monthly rainfall data was available for the period of January 1981 to December 2016; and here, summarized as mean monthly rainfall for periods of 10 years (Fig. 3). The periods between 1991–2000 and 2001–2010 reported lower mean monthly rainfall for both the long (in April–May–June, AMJ) and short rainy seasons (in October–November–December, OND) compared to other periods (1981–1990 and 2011–2016), regardless of the El-Nino rains of 1997 and 1998. For all periods during the long rains (AMJ), similar levels were observed, except in 1981–1990 and the peak of the rain season occurred during April except in 2011–2016 which had the peak in May. For the short rains (OND), the peak of the season occurred during the month of November (except in 1991–2000). Despite the differences observed over the study period, there was no shift in terms of the start and end of the long and the short rain seasons.

### Association between rainfall, bed nets and prevalence of malaria in Magoda and Mpapayu villages

**Fig. 4:**
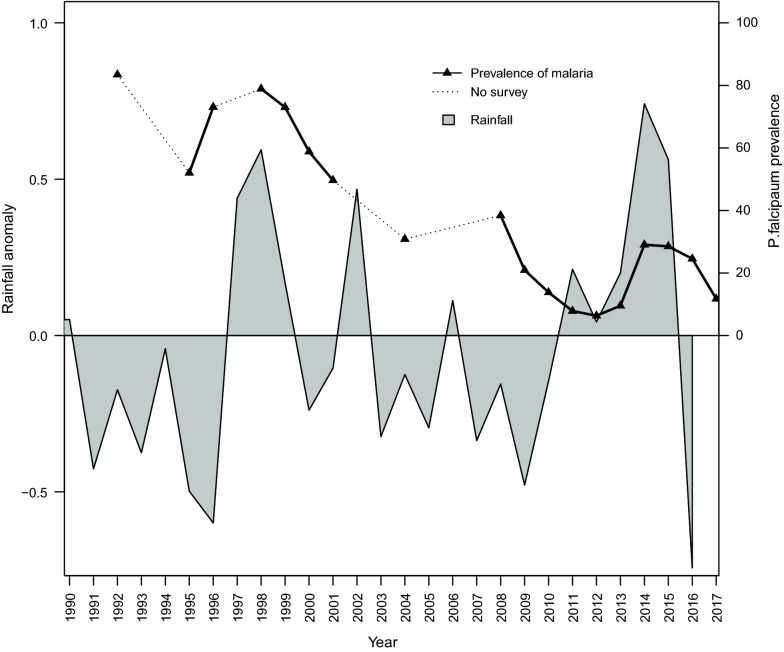
12-month weighted anomaly standardization precipitation (WASP) index for Muheza town from 1990 to 2016 and parasite prevalence in Magoda (1992–2017) and Mpapayu (1998–2017) villages

The 12-month weighted anomaly standardization precipitation (WASP) index (baseline = January 1981) showed a marked rainfall deficit in the periods between 1991–2000 and 2001 and 2010 (except in 1997–1999, 2001/02 and 2005/2006) and this seems to coincide with a significant decrease of malaria prevalence (Fig. 4). When explored by Poisson regression model (with robust standard errors) with the number of individuals who were positive for malaria parasites against bed net ownership, village, year of survey, rainfall anomaly and number of individuals surveyed as offset; the risk of being positive was decreasing across the years (Table 1). From 1992, the risk ratio slightly increased between 1999 and 2001, and thereafter decreased with the lowest risk ratio observed in 2013. Conversely, the risk of malaria parasite infections was significantly higher during the periods with positive rainfall anomalies; whereby a unit increase in rainfall anomaly had a corresponding increase of the risk of infection by a similar magnitude (RR = 2.79; 95% CI 2.32–3.51, P < 0.001). The risk of infections was higher among individuals who reported not to own bed nets compared to those who had bed nets (RR = 1.35, 95% CI 1.22–1.49, P < 0.001). For the two villages, the risk was lower in Mpapayu village (RR = 0.85, 95% CI 0.78–0.94, P = 0 0.001) compared to Magoda.

**Table 1:** Results from a Poisson regression model showing association between the risk of *Plasmodium falciparum* infections and rainfall anomaly adjusted for other factors

Variable	Risk ratio	95% CI	P > z
Rainfall anomaly: positive anomaly	2.793	(2.227–3.504)	< 0.001
Year of survey			
1999	1		
2000	1.208	(1.070–1.365)	0.002
2001	1.003	(0.858–1.173)	0.970
2004	0.626	(0.544–0.720)	< 0.001
2008	0.707	(0.595–0.841)	< 0.001
2009	0.549	(0.443–0.681)	< 0.001
2010	0.240	(0.177–0.327)	< 0.001
2011	0.096	(0.068–0.135)	< 0.001
2012	0.099	(0.082–0.121)	< 0.001
2013	0.072	(0.063–0.083)	< 0.001
2014	0.211	(0.167–0.266)	< 0.001
2015	0.233	(0.195–0.278)	< 0.001
Bed net ownership: no bed nets	1.350	(1.221–1.493)	< 0.001
Village: Mpapayu	0.849	(0.771– 0.936)	0.001

## Discussion

Previously, in the villages of Magoda and Mpapayu, Tanga region, Tanzania, a significant decline in the prevalence of *P. falciparum* infections was observed between 2000 and 2012 [19]. Parasite prevalence as low as < 10% was recorded in 2011 and 2012, and there were no individuals among under-fives from Mpapayu who had malaria parasites during the cross sectional survey performed in 2012. The villages had many interventions deployed during the period of 1998–2004 where the prevalence of *P. falciparum* declined, which would easily be ascribed to the interventions.

After the end of major research projects in the two villages in 2004, ITNs and LLINs were distributed to the study communities through different national initiatives including nets for pregnant women between 2006 and 2014, and mass distribution in 2011 and 2016 (NMCP, unpublished data). Moreover, the mobile clinic operating in the villages since the mid-1990s was in 2004 replaced with a dispensary, which offered medical services to all members of the community. Thus, the observed decline in malaria prevalence may be attributed to high coverage of ITNs/LLINs and improved case management at the dispensary, including deployment of effective anti-malarials (artemisinin-based combination therapy from 2007), as also described in the previous study [19]. It is, therefore, tempting to assume that the decline in malaria burden is closely related to the interventions as has been suggested for Zanzibar as well [35].

A large epidemiological malaria study conducted in Kenya suggests that the main factor responsible for the decline of malaria was the considerable use of SP in the management of uncomplicated malaria, when it replaced chloroquine as standard treatment in the early 2000’s [8]. Whether SP has caused a similar impact in the study area remains doubtful, as the decline began 1 year before the introduction of SP (in 2001) and high levels of SP resistance was already reported in these villages before SP was introduced, possibly related to previous mass drug administration of the antifolate drug combination, dapsone–pyrimethamine (Maloprim) in Magoda village in 1993–1994 [22, 36].

Alternatively, an entomological study performed in two villages of the same Tanga region located about 20 km from the present study observed a drastic decline in abundance of *An. gambiae* and *An. funestus*; from a sampling of 5382 *Anopheles* sp. in 2004 to merely a total of 14 mosquitoes in 2009 [37]. The cause of the almost complete disappearance of these mosquitoes is largely unknown as no major interventions in that study areas was done (coverage with bed nets did not exceed 27%). The study does suggest however, that part of the decline was associated with changes in patterns of monthly rainfall [37]. Whether the decline in *P. falciparum* prevalence observed between 2000 and 2012 in the present study sites was affected by lack of malaria vectors during this period is likely, and could possibly be related to the abnormalities in rainfall (amount and patterns), but unfortunately, no entomological surveys were performed during that study period.

From 2013 onwards, there has been a sustained re-emergence of malaria in the study area with an increase in parasite prevalence reaching 25% in 2014 and remaining relatively higher up to 2016 and a remarkable decline in 2017. Re-emergence of malaria in the study area occurred during a period where ITNs/LLINs are expected to work and a lack of protective effect of ITNs/LLINs somewhat reflects similar observations from a Kenyan study, where an increase in malaria prevalence was reported despite recent mass ITNs/LLINs distribution [8]. Although use of ITNs/LLINs among under-fives in Tanzania declined between 2012 and 2015 (from 72.7 to 54.5%), the level reported during this period was still high enough compared to the relatively low ITNs/LLINs coverage in 2004 (19.5%) and 2008 (32.7%) but with the highest decline in malaria burden (NMCP, unpublished data). Another potential contributing factor to resurgence of malaria might be development of resistance to insecticides used to impregnate the bed nets (even with LLINs), which has been reported in different parts of Tanzania including Muheza district [38–40].

The declining prevalence up to 2012 occurred in the period with overall negative rainfall anomaly suggesting that the decline in parasite prevalence and malaria burden reported in this area (and other parts of the country) was also possibly affected by the variability in the amount and pattern of rainfall directly affecting the *Anopheles* mosquitoes. These changes in prevalence occurred simultaneously with the interventions (high bed net coverage from 1998 in Magoda and 2001 in Mpapayu, and anti-malarial drugs) applied during the study period, indicating that multiple factors could be responsible for the observed trends. Conversely, the lack of changes in 2016 and a decline in prevalence in 2017 could possibly be attributed to the negative anomaly in 2016 and high bed net coverage (> 92%).

Thus, the decline in the prevalence of *P. falciparum* infections (and current resurgence) is most likely due to multiple factors including anti-malarial control interventions, but the exact contribution of the interventions is uncertain and possibly varies through time [2]. Additionally, factors such as climate, physical environment and socio-economic development are crucial and should be assessed in order to fully comprehend the changes in malaria epidemiology occurring in recent years. Further integrated surveillance is required to provide additional details which could be responsible for the decline as well as resurging transmission of malaria in North-eastern Tanzania as well as in other parts of Tanzania and sub-Saharan Africa [1], where malaria transmission patterns, socio-economic conditions; and climate are changing fast.

## Conclusions

A significant decline in the prevalence of *P. falciparum* infections observed up to 2012 was followed by a sustained resurgence of malaria, which is possibly associated with changes in the amount and pattern of rainfall in Muheza, apart from intensified malaria control. Thus, climate variability must be taken into consideration when relating control interventions to malaria prevalence. A sustained multi-factorial surveillance needs to be undertaken to monitor changes in malaria transmission and determine other factors which could be associated with continued transmission and resurgence of malaria in this and other area with a similar malaria epidemiological transition.

## Additional files

**Additional file 1.** Number of individuals sampled in CSS which were conducted in Magoda and Mpapayu between 1998 and 2016.

**Additional file 2.** Overall and age specific prevalence of *Plasmodium falciparum* detected by microscopy in the two villages of Magoda (1992–2017) and Mpapayu (1998 to 2017) in Muheza district north-eastern Tanzania.

## Abbreviations

AL: artemether–lumefantrine
AQ: amodiaquine
CSS: cross-sectional surveys
DANIDA: Danish International Development Agency
DDT: dichlorodiphenyltrichloroethane
ENRECA: Programme for enhancing research capacity in developing countries funded by DANIDA
ITNs: insecticide treated bed nets
LLINs: long-lasting insecticidal nets
MoHCDGEC: Ministry of Health, Community Development, Gender, Elderly and Children
MRCC: Medical Research Coordinating Committee
RDTs: rapid diagnostic tests
NIMR: National Institute for Medical Research
NMCP: National Malaria Control Programme
SP: sulfadoxine/pyrimethamine
TMA: Tanzania Meteorological Agency
WASP: 12-months weighted anomaly standardized precipitation index
WHO: World Health Organization
