# Supplementary material for: Trends of Plasmodium falciparum prevalence in two communities of Muheza district North-eastern Tanzania: correlation between parasite prevalence, malaria interventions and rainfall in the context of re-emergence of malaria after two decades of progressively declining transmission
Source: Malar J. 2018 Jul 6;17:252. doi: 10.1186/s12936-018-2395-1 (PMC6034219; doi:10.1186/s12936-018-2395-1)
Supplement: Supplementary file 1 — Additional file 1. Number of individuals sampled in CSS which were conducted in Magoda and Mpapayu between 1998 and 2016. [file 12936_2018_2395_MOESM1_ESM.docx]

**Additional file 1: Number of individuals sampled in CSS which were conducted in Magoda and Mpapayu between 1998 and 2016.**

| **Month-Year** | **Magoda** | | | **Mpapayu** | | |
| --- | --- | --- | --- | --- | --- | --- |
|  | **N** | **Mean age (range)** | **<5 years (%)** | **N** | **Mean age (range)** | **<5 years (%)** |
| October 1998 | 181 | 2.5(0.2,4.9) | 100.0 | 78 | 2.6(0.1,5.0) | 100.0 |
| December 1999 | 383 | 6.9(1.0,19.6) | 40.7 | 232 | 6.8(1.0,19.6) | 39.2 |
| November 2000 | 352 | 6.8(1.0,19.9) | 37.8 | 226 | 7.3(1.0,19.6) | 34.5 |
| April/May & August  September 2001 | 352 | 6.4((1.0,19.7) | 42.3 | 202 | 7.2(1.0,19.7) | 39.1 |
| April/May & August   - October 2004 | 392 | 7.0(1.0,19.7) | 36.2 | 237 | 6.8(1.0,19.7) | 38.8 |
| May 2008 | 422 | 7.4(0,19.8) | 41.5 | 268 | 8.3(0.3,19.5) | 36.6 |
| May/June 2009 | 512 | 8.3(0.4,19,9) | 33.4 | 267 | 8.0(0.4,19.5) | 38.2 |
| May/June 2010 | 480 | 8.2(0.4,19.9) | 33.8 | 273 | 8.1(0.1,19.9) | 34.8 |
| May 2011 | 483 | 9.0(0.5,20.3) | 26.5 | 258 | 8.6(0.4,19.8) | 29.1 |
| June 2012 | 460 | 8.7(0.1,19.9) | 26.3 | 253 | 8.5(0.5,19.7) | 26.9 |
| May 2013 | 458 | 8.6(0.2,19.3) | 29.0 | 254 | 8.1(0.1,19.4) | 29.5 |
| June 2014 | 449 | 8.3(0.5,19.9) | 30.5 | 247 | 8.9(0.5,19.8) | 27.1 |
| May 2015 | 453 | 8.9(0.7,19.5) | 24.9 | 251 | 8.8(1.1,19.0) | 21.5 |
| June 2016 | 414 | 8.8(0.5,19.9) | 28.5 | 251 | 9.5(0.5,18.6) | 16.7 |
| May/June 2017 | 465 | 8.6(0.4, 20.8) | 13.3 | 288 | 9.3(0.2,19.9) | 11.1 |

CSS = Cross-sectional survey, N = Number of individuals included in the CSS
