# Supplementary material for: Trends of Plasmodium falciparum prevalence in two communities of Muheza district North-eastern Tanzania: correlation between parasite prevalence, malaria interventions and rainfall in the context of re-emergence of malaria after two decades of progressively declining transmission
Source: Malar J. 2018 Jul 6;17:252. doi: 10.1186/s12936-018-2395-1 (PMC6034219; doi:10.1186/s12936-018-2395-1)
Supplement: Supplementary file 2 — Additional file 2. Overall and age specific prevalence of Plasmodium falciparum detected by microscopy in the two villages of Magoda (1992–2017) and Mpapayu (1998 to 2017) in Muheza district north-eastern Tanzania. [file 12936_2018_2395_MOESM2_ESM.docx]

**Additional file 2: Overall and age specific prevalence of *Plasmodium falciparum* detected by microscopy in the two villages of Magoda (1992 -2017) and Mpapayu (1998 to 2017) in Muheza district north-eastern Tanzania**

| **Village** | **Age group** | **1992** | **1995** | **1996** | **1997** | **1998*** | **1999** | **2000** | **2001** | **2004** | **2008** | **2009** | **2010** | **2011** | **2012** | **2013** | **2014** | **2015** | **2016** | **2017** |
| --- | --- | --- | --- | --- | --- | --- | --- | --- | --- | --- | --- | --- | --- | --- | --- | --- | --- | --- | --- | --- |
| Magoda | 0-4yrs | 83.6 | 86.0 | - | - | 76.0 | 61.9 | 48.1 | 38.3 | 26.1 | 26.3 | 11.1 | 11.7 | 3.9 | 3.3 | 6.8 | 23.4 | 17.0 | 18.6 | 6.6 |
|  | 5-9rys | 91.4 | 98.7 | - | - | - | 78.1 | 61.7 | 62.3 | 41.5 | 55.6 | 24.2 | 15.5 | 9.9 | 6.7 | 9.4 | 32.0 | 32.3 | 29.6 | 15.7 |
|  | 10-19yrs | 78.2 | 86.8 | - | - | - | 57.4 | 43.9 | 38.6 | 32.8 | 57.7 | 31.6 | 17.6 | 12.9 | 10.0 | 18.2 | 36.0 | 39.5 | 31.1 | 16.8 |
|  | **Overall** | **83.5** | **77.0** | **62.2** | **79.0** | **76.0** | **67.9** | **53.7** | **48.3** | **34.4** | **44.1** | **22.5** | **15** | **9.5** | **7.2** | **12.1** | **20** | **31.4** | **27.1** | **13.3** |
| Mapapayu | 0-4yrs | - | - | - | - | 76.5 | 80.2 | 65.4 | 41.8 | 18.5 | 17.4 | 7.8 | 4.2 | 1.4 | - | 1.3 | 10.5 | 14.8 | 11.9 | 7.0 |
|  | 5-9rys | - | - | - | - | - | 89.4 | 77.2 | 66.7 | 29.5 | 40.9 | 15.9 | 9.0 | 2.7 | 3.2 | 4.9 | 26.9 | 22.7 | 17.4 | 15.4 |
|  | 10-19yrs | - | - | - | - | - | 53.6 | 46.8 | 44.4 | 27.5 | 33.7 | 30.2 | 21.0 | 8.0 | 9.8 | 7.2 | 35.3 | 27.5 | 26.5 | 9.4 |
|  | **Overall** | - | - | - | - | **76.5** | **81.5** | **66.8** | **52.0** | **24.9** | **29.5** | **18.0** | **11.7** | **4.6** | **4.7** | **4.7** | **25** | **23.1** | **20.7** | **11.1** |

*Only under-fives were enrolled in the survey of 1998 in both villages.
